# Inhibition of signal peptidase complex expression affects the development and survival of *Schistosoma japonicum*

**DOI:** 10.3389/fcimb.2023.1136056

**Published:** 2023-03-03

**Authors:** Wen-Bin Yang, Fang Luo, Wei Zhang, Cheng-Song Sun, Cong Tan, An Zhou, Wei Hu

**Affiliations:** ^1^ State Key Laboratory of Genetic Engineering, Ministry of Education Key Laboratory of Contemporary Anthropology, Human Phenome Institute, Fudan University, Shanghai, China; ^2^ Ministry of Education Key Laboratory for Biodiversity Science and Ecological Engineering, Department of Microbiology and Microbial Engineering, School of Life Sciences, Fudan University, Shanghai, China; ^3^ The State Key Laboratory of Reproductive Regulation and Breeding of Grassland Livestock, College of Life Sciences, Inner Mongolia University, Hohhot, China; ^4^ Central Laboratory, Anhui Provincial Institute of Parasitic Diseases, Anhui, China

**Keywords:** *S. japonicum*, SPC, *SPC25*, RNAi, development, reproduction

## Abstract

**Background:**

Schistosomiasis, the second most neglected tropical disease defined by the WHO, is a significant zoonotic parasitic disease infecting approximately 250 million people globally. This debilitating disease has seriously threatened public health, while only one drug, praziquantel, is used to control it. Because of this, it highlights the significance of identifying more satisfactory target genes for drug development. Protein translocation into the endoplasmic reticulum (ER) is vital to the subsequent localization of secretory and transmembrane proteins. The signal peptidase complex (SPC) is an essential component of the translocation machinery and functions to cleave the signal peptide sequence (SP) of secretory and membrane proteins entering the ER. Inhibiting the expression of SPC can lead to the abolishment or weaker cleavage of the signal peptide, and the accumulation of uncleaved protein in the ER would affect the survival of organisms. Despite the evident importance of SPC, *in vivo* studies exploring its function have yet to be reported in *S. japonicum*.

**Methods:**

The *S. japonicum* SPC consists of four proteins: *SPC12, SPC18, SPC22* and *SPC25*. RNA interference was used to investigate the impact of SPC components on schistosome growth and development *in vivo*. qPCR and *in situ* hybridization were applied to localize the *SPC25* expression. Mayer’s carmalum and Fast Blue B staining were used to observe morphological changes in the reproductive organs of dsRNA-treated worms. The effect of inhibitor treatment on the worm’s viability and pairing was also examined *in vitro*.

**Results:**

Our results showed that RNAi-SPC delayed the worm's normal development and was even lethal for schistosomula *in vivo*. Among them, the expression of *SPC25* was significantly higher in the developmental stages of the reproductive organs in schistosomes. Moreover, *SPC25* possessed high expression in the worm tegument, testes of male worms and the ovaries and vitellarium of female worms. The SPC25 knockdown led to the degeneration of reproductive organs, such as the ovaries and vitellarium of female worms. The *SPC25* exhaustion also reduced egg production while reducing the pathological damage of the eggs to the host. Additionally, the SPC-related inhibitor AEBSF or suppressing the expression of *SPC25* also impacted cultured worms’ pairing and viability *in vitro*.

**Conclusions:**

These data demonstrate that SPC is necessary to maintain the development and reproduction of *S. japonicum*. This research provides a promising anti-schistosomiasis drug target and discovers a new perspective on preventing worm fecundity and maturation.

## Introduction

Schistosomiasis, a neglected tropical disease caused by the parasitic flatworm *Schistosoma japonicum*, affects more than 230 million people worldwide (Barnett, 2018). This trematode has a complicated life cycle involving asexual reproduction within molluscan intermediate hosts and sexual reproduction within definitive mammalian hosts. As a striking parasite, *S. japonicum* can last for decades in the host, and every pair of worms can possess a prodigious egg output of 1,000 ~ 2,200 per day (Cheever et al., 1994; Kuntz et al., 2007; Schwartz and Fallon, 2018). The prodigious egg production resulting from the pairing of female worms is swept into the circulation and then deposited or trapped in the host organ, which can cause inflammatory reactions and disturb normal function, such as liver fibrosis and hypertension (Hambrook and Hanington, 2020; Takaki et al., 2020). Thus, worm transmission and disease pathogenesis were both completed by the eggs. Both host pathological disruption and disease transmission will be prevented if the parasites can be killed or deprived of oviposition capacity. However, only one drug (praziquantel) is currently effective in treating schistosomiasis. This medication does not prevent reinfection and has little effect on the schistosomula (Brindley and Sher, 1987; Brindley et al., 1989). Besides, the emergence of drug-resistant parasites also emphasizes the urgent need for novel therapeutics (Kuntz et al., 2007; Le Clec'h et al., 2021). Although considerable research has been employed over the years, the extremely complex life cycle and the technological barriers to genetic manipulation of *Schistosoma* make it challenging to find an effective gene as the antischistosomal drug target (Kuntz et al., 2007; Han et al., 2009; Cioli et al., 2014; Eyayu et al., 2020).

Detailed dynamic transcriptome studies indicated that a subset of highly expressed mRNA in the development stage of male-female worm pairing has also been characterized as being related to protein biosynthesis (Wang et al., 2017). The comparative analysis of single-sex and double-sex infections of *S. japonicum* also showed that the differentially expressed proteins of paired females were mainly enriched in the bioinformatics related to protein synthesis (Li et al., 2020). Recent research also revealed that a specialized ribosomal protein expression profile after pairing was upregulated to regulate the protein synthesis process for sexual maturation (Sun et al., 2015). The increasing body of evidence suggests that protein synthesis-related genes involved in parasite development, maturation, and reproduction can serve as an essential group of drug targets to block the spread of schistosomiasis (Avelar et al., 2019; Zeng et al., 2022). Consistent with this notion, the results of a large-scale RNAi screen of *S. mansoni* revealed that many gene phenotypes affecting neuromuscular function, tissue integrity, stem cell maintenance, and parasite survival are associated with protein synthesis processes (Guidi et al., 2015; Ittiprasert et al., 2019; Wang et al., 2020).

The signal peptidase complex (SPC) was used in the translocation of proteins into the endoplasmic reticulum (ER) by cleaving the signal peptide sequence (SP) of secretory and membrane proteins (Paetzel et al., 2000; Haase Gilbert et al., 2013). In *S. cerevisiae*, the composition of SPC has four proteins: *Sec11*, *Spc1p*, *Spc2p* and *Spc3p*. Previously, studies have shown that depletion of *SPC3* and *Sec11p* elicits the accumulation of uncleaved protein in the ER. It also confirmed that *SPC3* and *Sec11p* are responses for the viability of yeast cells, while Spc1p and Spc2p are essential but not necessarily for *S. cerevisiae* growth. (Mullins et al., 1996; Meyer and Hartmann, 1997). In *Drosophila*, *Spase12* depletion was embryonic lethal and resulted in developmental defects in tissues, indicating that *Spase12* could regulate cell viability, development, and differentiation (Haase Gilbert et al., 2013). In mammals, *SPC18*, *SPC21*, *SPC22/23*, *SPC12* and *SPC25* comprise the SPC, and all five subunits are essential to the proper release of the secreted protein and localization of membrane proteins synthesized with cleaved signal peptides (Shelness et al., 1993). ER-related protein is essential in eukaryotic cells and is particularly critical in fast-growing cells (Kalies and Romisch, 2015). It has been confirmed that the protein synthesis process can serve as a target for potential cancer drugs and bacterial virulence factors (Harbut et al., 2012). In yeast and mammalian cells, the function of signal peptidases has been elucidated. However, there is little information about its role in multicellular organisms, for example, in *S. japonicum*. Similar to cancer cells, the ovary, vitellarium and testis of *S. japonicum* have cells with high proliferative activity, requiring more protein synthesis to maintain the cell proliferation and differentiation (Buszczak et al., 2014; do Patrocinio et al., 2020). Given the importance of SPC in other species and its expression pattern in *S. japonicum*, we hypothesized that SPC might be essential to worm survival and fecundity.

To examine this hypothesis, we contributed to characterizing SPC molecular function in *S. japonicum*. In this study, we employed dsRNA-mediated RNA interference (RNAi) *in vivo* to characterize gene function. We observed that SPC played an essential role in the development and survival of schistosomula. Further studies confirmed that *SPC25* had higher expression levels in the reproductive organs of worms. In addition, this gene was shown to be essential for maintaining the normal morphology of worm reproductive organs and for the oviposition of the female worm. These findings provided insights into genes that regulated the development and egg production of *S. japonicum* and revealed a novel class of antischistosomal candidates for therapeutic opportunities.

## Materials and methods

### Parasites and animals

The *S. japonicum* (Anhui isolate) strain used in this study was obtained from *Oncomelania* Anhui snails provided by the pathobiology laboratory of the National Institute of Parasitic Diseases, Chinese Center for Disease Control and Prevention, Shanghai. Six-week-old female Kunming mice purchased from Shanghai Animal Center, Chinese Academy of Sciences (Shanghai, China), were infected with *S. japonicum*. Schistosome-infected Kunming mice were euthanized at 30 or 42 days post-infection (dpi) by carbon dioxide asphyxiation. And then, parasites were isolated from the infected mice by hepatic-portal perfusion using a saline solution containing heparin sodium (Li et al., 2018).

### Amplification and sequencing

Total schistosome RNA was extracted using TRIzol (Takara, Japan) from different developmental stages, sexes and regions of worms. The extracted RNA was quantified using a Nanodrop ND2000C Spectrophotometer (Nanodrop Technologies, Wilmington, DE). The cDNA was synthesized from 1000 ng of RNA using the PrimeScript RT reagent Kit with gDNA Eraser (Takara, Japan) according to the manufacturer’s instructions. Primers to amplify target sequences were designed using the Primer 5 program described in **Table 1** (Li et al., 2018). Target fragments were amplified from *S. japonicum* cDNA and sequenced by Huajin Biotech Co. Ltd (Shanghai, China).

**Table 1:** List of primers used in this study.

	Forward Primer	Reverse Primer
SPC12-dsRNA	TTTACGTTACGGTTTGACG	CAACGACTGCTGTGAGTA
SPC12-qRNA	TATACTCACAGCAGTCGTTG	TAGTCTCTTCCGTATACCAG
SPC18-dsRNA	CGACTTTAAGCGTATGAACA	AACCAATCTTCACCAGGAG
SPC18-qRNA	CTTCATTCCTTATATCGGTCAG	GTAGCAAGTAGATACTCATAGC
SPC22-dsRNA	TTACTGACCATCACTCTCAC	TTACTGACCATCACTCTCAC
SPC22-qRNA	GCAACGATAATGTCACCTTA	AACGAGTGATCGCCAATG
SPC25-dsRNA	AGAAGTTACAGCCAATAAGTG	ATCTAAACCCGTCTTGTCTT
SPC25-qPCR	CTGATTCGTGGACAGCAT	CACAGAAGATACCTTGATACTC
SPC25-probe	TAATACGACTCACTATAGGGAGAGAAGTTACAGCCAATAAGTG	TCTAAACCCGTCTTGTCTT

The full-length sequences of *SPC12* (Sjc_0006936), *SPC18* (Sjc_0009443), *SPC22* (Sjc_0006565) and *SPC25* (Sjc_0003692) were obtained from the *S. japonicum* database and NCBI. The primers were designed for fragments for dsRNA synthesis, riboprobe of RNA and RT-qPCR using the Primer 5 program described in **Table 1** (Li et al., 2018). Fragments of green-fluorescent protein (GFP, KU312311.1) were used as the negative control in the following RNAi experiments.

### Gene expression detection

#### Different time points

The samples collected at 14, 16, 18, 20, 22, 24, 26 and 28 dpi of both genders of worms were used to profile the levels of the target-gene transcript at different time points among the paring stages of *S. japonicum* for qRT-PCR analysis (Guidi et al., 2015).

#### Different organs

In order to detect the expression of target genes in different tissues, the female and male worm was dissected into three parts (anterior, ovary and vitellarium) and two parts (testis and posterior), respectively, according to the previous method (Hahnel et al., 2013). The parasites were collected from mice after 28 dpi (Zhu et al., 2016).

#### Gene knockdown detection

For detection of the knockdown level of the target gene, the harvested six pairs of worms (males and females were separated) were used to extract total RNA for qRT-PCR analysis. The qRT-PCR analysis was carried out in triplicates technical replicates using strictly designed primers. The gene encoding 26S proteasome non-ATPase regulatory subunit 4 gene (*Sj*PSMD) was used as the endogenous standard for each sample. The 2^-ΔCt^ method was used to calculate the relative expression (Li et al., 2018).

### RNA interference

#### dsRNA synthesis

Special specific primers with the T7 promoter were designed to amplify a PCR product of ~500 bp for the target gene. PCR products were used to generate the dsRNA of the target gene using the MEGAscript T7 transcription kit (Catalogue No. AM1334, Ambion) following the manufacturer’s guidelines. End products were checked with agarose gel electrophoresis (Collins and Collins, 2016; Wang et al., 2017). Moreover, a non-specific green fluorescent protein (GFP) sequence was successfully used to produce dsRNA, serving as a negative control (Li et al., 2018).

#### Schistosomula silencing *in vivo*

**Figure 1:**
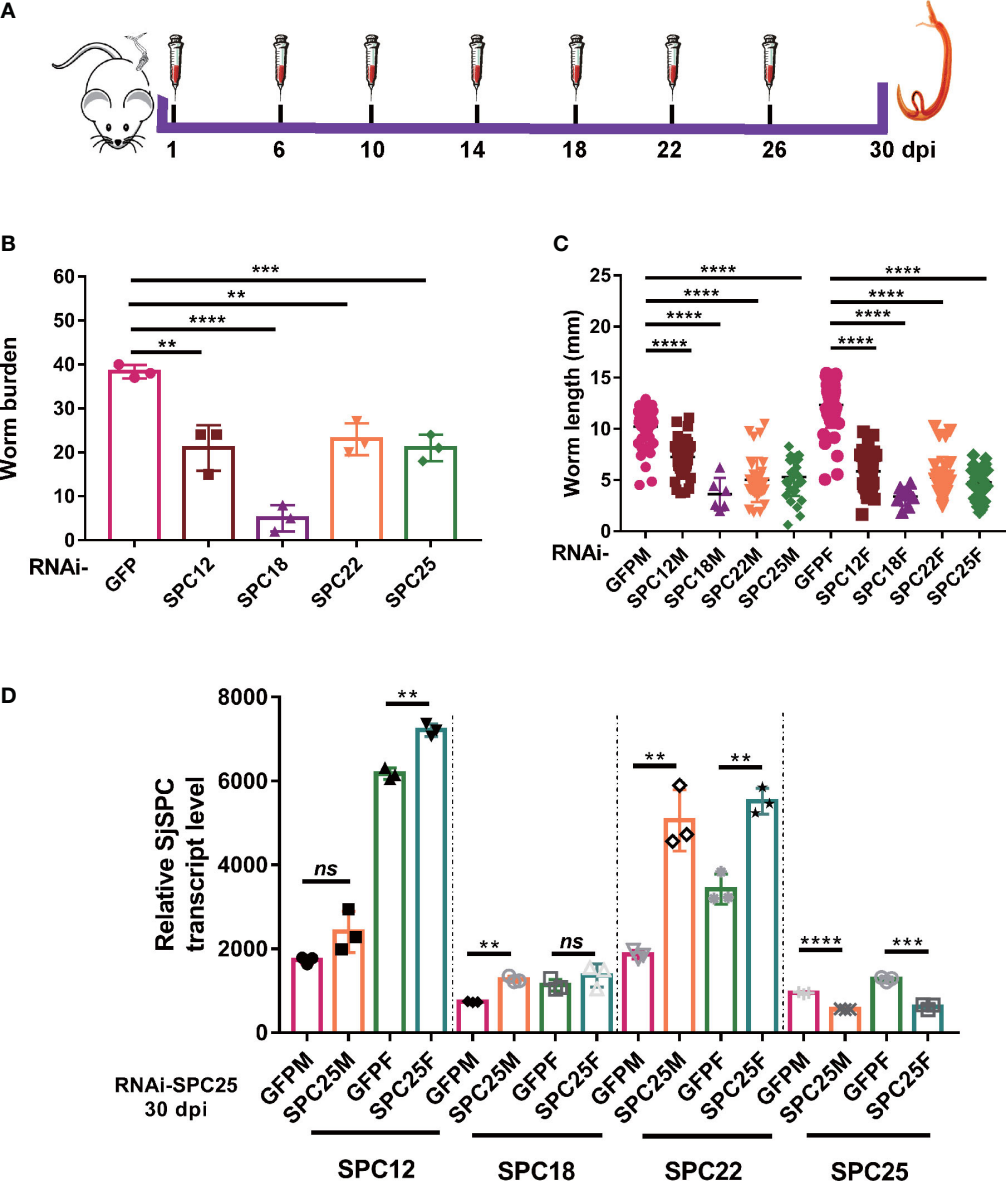
Suppressing the expression of SPC elements dramatical impairing the development and leading to the death of schistosomula. **(A)** Schematic diagram of the gene interference process of schistosomula *in vivo*. **(B)** Assessment of worm burden of dsRNA-mediated RNA effects. **(C)** Evaluation of worm length recovered from mice at 30 dpi. And worm length data were obtained from all worms recovered in three mice. **(D)** The expression level of *SPC25* and other components of SPC in the RNAi-SPC25 worms. Each group had three biological replicates (three mice), and the data were shown as means ± SEM. **p < 0.01, ***p < 0.001, ****p < 0.0001, ns, not significant, t-test.

After receiving 60 cercariae, 15 Kunming mice were divided into five groups at random: *SPC12*, *SPC18*, *SPC22*, *SPC25* and GFP dsRNA treatment group. Subsequently, each mouse was injected with 10 μg (25 pmol) of dsRNA dissolved in 0.2 ml saline buffer (0.7% NaCl) into the tail vein at the same dose on 1, 6, 10, 14, 18, 22, 26 dpi, respectively (**Figure 1A**). This injected dose ensures sustained suppression of target gene expression in *S. japonicum*. Worms were collected at 30 dpi, as described in a previous study (Li et al., 2018).

#### Adult worm silencing *in vivo*

**Figure 2:**
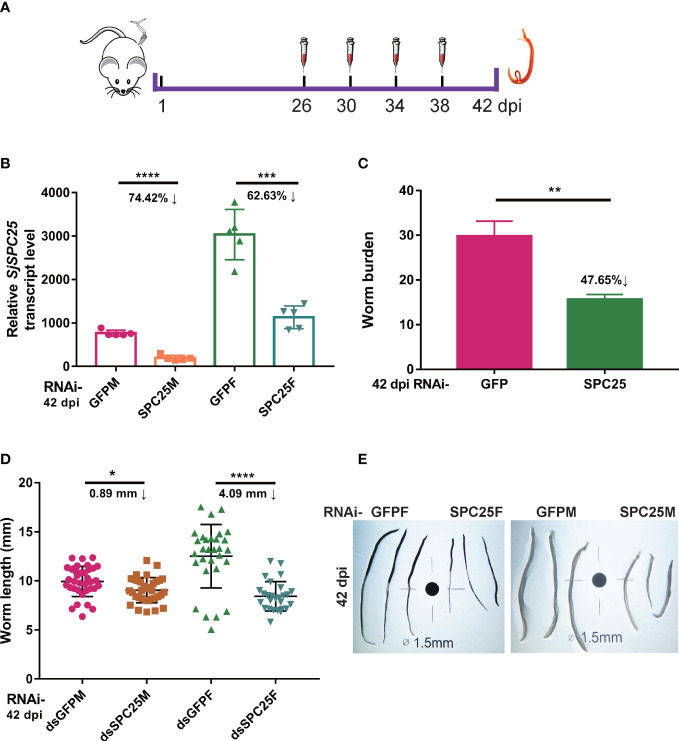
Effection on development and survival of adult *S. japonicum.* **(A)** Schematic diagram of the gene interference process of adult worms *in vivo*. **(B)** Efficacy assessment of dsRNA-mediated *SPC25* knockdown of worm collected at 42 dpi by qRT-PCR. **(C)** The number of worms collected at 42 dpi from the dsGFP- or dsSPC25-injected mouse. Each group had five biological replicates. **(D)** Measurement of all worm lengths recovered from five mice in each group at 42 dpi. The data were shown as means ± SEM. **(E)** Comparison of the length of worms collected at 42 dpi. *p < 0.05, **p < 0.01, ***p < 0.001, ****p < 0.0001, t-test.

Two groups (RNAi-SPC25 and RNAi-GFP) of 6-week-old female mice infected with 40 ± 2 freshly shed cercariae, with five mice per group. This dose of cercariae infection ensures that no mortality occurs in the experimental mice at 42 dpi. At 26 dpi, 10 μg (25 pmol) of dsRNA was injected into the corresponding groups *via* the tail vein for the first time, and then additional injections were given every four days (days 26, 30, 34 and 38 post-infection) until 42 dpi (**Figure 2A**). Harvested parasites were treated similarly, as described above.

#### RNAi experiments *in vitro*

At 37°C and 5% CO_2_, 6 freshly perfused pairs of worms were treated with 10 μg/ml dsRNA in 3 ml medium in a 12-well plate (DMEM, 10% fetal bovine serum, 1% HEPES buffer, 2% penicillin/streptomycin solution). The worms were kept in culture for observation for up to 30 days. The culture was refreshed every other day, and fresh dsRNA was added on days 1, 3, 7, 11, 15, 19, 23 and 27, respectively. During this time, worms were observed daily for motility and viability (Wang et al., 2019; Wang et al., 2020). Samples of treated parasites were collected to access gene knockdown 15 days and 30 days after the dsRNA treatment.

### Schistosoma imaging

#### Worm length

For quantitation of parasite growth, the dsRNA-treated worms were fixed with AFA (95% alcohol, 3% formaldehyde, and 2% glacial acetic acid) and photographed under a Nikon SMZ445 dissecting microscope at 4 X. Then, the captured digital micrographs of worm length and diameter were analyzed using ImageJ software (https://imagej.nih.gov/ij/) and GraphPad Prism 6.0 (Shen et al., 2017).

#### *In situ* hybridization

In order to determine the *SPC25* mRNA localization, *in situ* hybridization was performed in *S. japonicum*. The WISH probe was labeled with digoxin and then purified according to the method provided by the kit (Ambion, Catalogue No. AM1908). Worms were harvested from the infected mice after 30 dpi. Separated female and male worms were fixed with 4% paraformaldehyde dissolved in PBSTX (1X PBS, 0.3% TritonX-100). Subsequently, the fixed parasites were used to present the location of the *SPC25* transcript using the WISH method according to what was previously described (Collins et al., 2016; Wang et al., 2019; Wendt et al., 2020).

#### Mayer’s carmalum

To further evaluate the impact on the worm reproductive organ, the collected worms were stained with Mayer’s carmalum. Briefly, recovered worms were fixed with AFA, and then treated with glacial acetic acid and dehydration in an ethanol gradient. The samples were dyed with 2.5% alumcarmine for 5 minutes, incubated in 5% aluminium potassium sulfate for 15 minutes, and then destained in 70% acidic ethanol. Parasites dehydrated through an ethanol gradient were transferred to methyl salicylate for clarification and mounted with Canada Balsam on glass slides (Shen et al., 2017; Li et al., 2018). Morphometric observations were performed on male and female worms under a light microscope (Nikon 80i) and Laser Scanning Confocal Microscopy (LSCM, Nikon A1-Ni).

#### Fast Blue B

Fast Blue B staining could be visualized the developmental level of the vitellarium due to the characteristic of strong affinity with the vitelline droplets within the mature vitellarium of female worms. The separated female worms were fixed in 75% alcohol. Then, the samples were washed, stained with 1% Fast Blue B for 1 minute, dehydrated, clarified in ethanol with dimethylbenzene (1:1), and embedded in Canada Balsam. Images of the staining of vitelline cells were collected with a Nikon 80i microscope (Wang et al., 2017).

### Liver egg burden, hatchability and tissue histopathology

To provide further evidence of the role of *SPC25* in worm fecundity, the worm eggs laid by knockdown parasites were numbered in mouse livers. The liver was recovered from each mouse and then divided into three parts to conduct three investigatory components: one was used for the depositing eggs number calculation, one for egg hatching rate assessment, and another part for granuloma analysis according to the technique previously described (Anderson et al., 2019; Avelar et al., 2019; Cheng et al., 2020).

#### Egg burden

For egg counting, individuals from the right liver lob were weighed and homogenized into homogenate suspensions, which were then digested with 10 ml 5% NaOH solution overnight at 37°C. The degree of egg burden was determined by microscopic observation based on counting the number of eggs on 7 slides prepared from each liver homogenate, as previously described (Avelar et al., 2019; Cheng et al., 2019).

#### Egg hatching rate

After verifying the change in liver egg burden, the eggs laid by dsRNA-treated worms were isolated and purified to calculate the hatching rate. To do this, about 200 eggs were divided among 24-well culture plates, and every 30 minutes for up to 4 hours, the number of miracidia that hatched was counted using a light microscope. The miracidia were further fixated with iodine and collected to count as previously described (Wang et al., 2019).

#### Tissue histopathology

The collected left liver from each mouse was fixed with paraformaldehyde overnight. After dehydration, the tissues were cleared with xylene and embedded in paraffin wax. 4 μm thick sections were prepared and the tissues were stained with hematoxylin and eosin (HE) and Masson staining to evaluate the egg granuloma formation and the deposition of collagen fibers (Luo et al., 2022). At last, the sections were observed under a microscope (Cheng et al., 2020; Hu et al., 2020).

### Inhibitor treatment of parasites

6 freshly perfused pair worms were cultured in wells of 12 well plates in 3 ml media. Cultures were supplemented with AEBSF inhibitor (Selleck, China) dissolved in DMSO (Conseil et al., 1999). Different concentrations (10 μM, 5 μM, 2.5 μM, 1 μM) of AEBSF inhibitor were added to the wells and 0.1% DMSO to controls. Cultures and inhibitor were renewed every other day. Three independent biological replicates were used in this experiment.

### Statistical analysis

Data are expressed as the mean ± SEM for at least three independent experiments. Different groups of dsRNA treatment were tested for significant differences using Student’s t-test, where *P*-value ≤ 0.05 was considered statistically significant (Luo et al., 2022). Statistical analyses were performed with GraphPad Prism 6.0.

## Results

### Silencing of SPC elements led to developmental delay and death in schistosomula

To explore the function of SPC pathway elements in the growth and development of parasites, we used RNAi technology to interfere with target genes. Six injections of dsRNA into mice *via* the tail vein were used to achieve long-term sustained repression of SPC elements (**Figure 1A**). After 30 dpi, adult worms were perfused from the hepatic portal system. SPC components knockdown resulted in a significant decrease in the number of adult worms (44.57% for RNAi-SPC12, 86.28% for RNAi-SPC18, 39.99% for RNAi-SPC22, and 45.21% for RNAi-SPC25) recovered from infected mice when compared to unspecific control (**Figure 1B**). The experimental results confirmed that most of the SPC RNAi-treated worms failed to establish infection in the host. Hence, SPC components are essential for schistosomula to establish infection in hosts.

At the same time, we also found that the growth of worms in the RNAi-SPC component group was significantly delayed. The length of worms showed that schistosomula recovery from the RNAi-SPC25 group in both female and male worms (5.16 ± 0.2781 mm and 4.797 ± 0.2438 mm, respectively) was significantly smaller than that of dsGFP-treated parasites (10.21 ± 0.2083 mm and 12.30 ± 0.2711 mm, respectively). Compared with the control group, worm length was inhibited in other groups, among which the lengths of *SPC12* male and female worms were shortened by 29.13% and 52.63%, *SPC18* was shortened by 54.57% and 72.40%, *SPC22* was shortened by 50.86% and 57.51%, respectively (**Figure 1C**). Thus, we reasoned that SPC component*s* appear to be essential for schistosome growth and survival *in vivo*.

Analysis of transcriptome data using different stages of *S. japonicum* in the laboratory showed that SPC elements have the same expression pattern (Wang et al., 2019). The expression of SPC elements was relatively constant in males and significantly up-regulated in females at 20-26 dpi (**Figure S1A**). This time is a critical period that the reproductive system develops rapidly in the female worm. In addition, the results of *S. japonicum* single-cell sequencing showed that the SPC element had the similar cellular localization (unpublished data). The four SPC elements had higher expression in the tegument cell populations and germ cell populations of male and female worms (**Figure S1B**). Based on the above results, we can infer that SPC elements have similar biological functions.

In order to gain a more profound understanding of the explicit function of SPC elements in *S. japonicum*, we selected one of the SPC components for further research. Previous studies confirmed that the RNAi-SPC18 group had a worm reduction rate of 86.28%, the number of worms recovered from this experimental group could not be sufficient for the requirement of subsequent experiments. Therefore, we selected *SPC25* for further investigation.

Though seven times with dsRNA injection *via* tail vein, *SPC25* mRNA levels, compared to the RNAi-GFP group, were significantly exhausted in both genders, ranging from 41.39% to 51.12% reduction in male and female worms at 30 dpi, respectively (**Figure 1D**). The results also showed that the expression of other components of SPC was upregulated after the knockdown of *SPC25* (**Figure 1D**). This result shows SPC25-specific dsRNA is effective for *SPC25* silencing during the development of worms *in vivo*.

### Expression pattern and localization of the *SPC25* transcript in *S. japonicum*

#### Gene-expression profiles at different developmental time points

**Figure 3:**
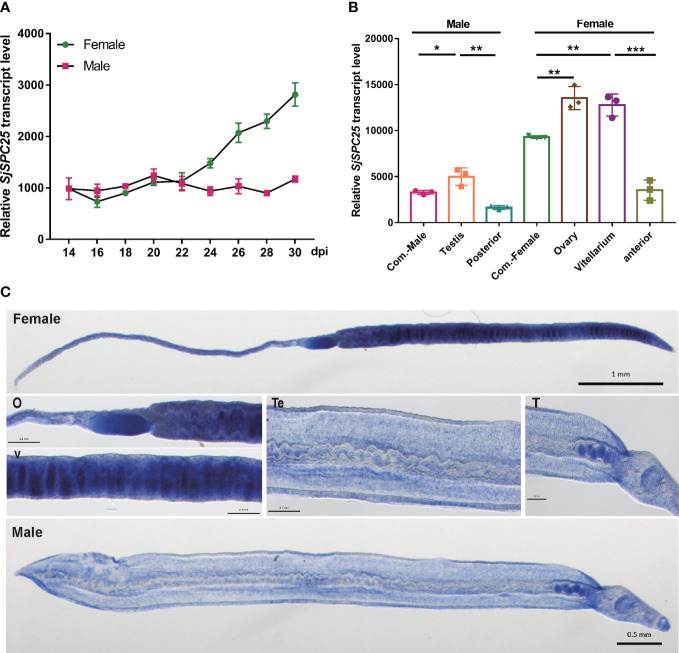
Profiles of the *SPC25* transcript level. **(A)** *SPC25* mRNA expression level in male and female worms at different development stages of *S.japonicum*. **(B)** The transcript level of *SPC25* at isolated reproductive organs. All experiments were performed in triplicate with three biological replicates, and each group’s data were shown as means ± SEM. **(C)** *In situ* hybridization analysis of *SPC25* in the male and female worms. Te, tegument; T, testis; O, ovary; V, vitellarium. *p < 0.05, **p < 0.01, ***p < 0.001, t-test.

The expression profile of *SPC25* was measured at different developmental time points in the male and female worms of *S. japonicum* using SYBR real-time RT-PCR. The results demonstrated that the *SPC25* mRNA had distinctive expression patterns between male and female parasites. We found that *SPC25* was highly expressed in the female worm and gradually increased after worm pairing. Moreover, after the pairing of male and female worms, its expression level was relatively stable and slightly decreased in the male (**Figure 3A**). These results were consistent with the early study about the detailed dynamic transcriptome (Wang et al., 2017).

#### Gene-expression levels in the isolated gonad

To further corroborate the association between *SPC25* and worm development, we checked the abundance of the *SPC25* gene transcript in different dissected organs of a worm. The results suggested that *SPC25* mRNA expression was significantly higher in the ovary and vitellarium of adult female *S. japonicum*. It was also predominantly expressed in the testes compared with the posterior in the male schistosomes (**Figure 3B**). We reasoned that *SPC25* might be a crucial gene for supporting the further development of reproductive organs.

#### Whole-mount *in situ* hybridization

Whole-mount *in situ* hybridization (WISH) experiments were carried out to clarify the relationship between *SPC25* and the reproductive organs of *S. japonicum*. We observed the transcriptional signals of *SPC25* in the ovary and vitellarium of female worms and the tegument and testes of male worms (**Figure 3C**), indicating that *SPC25* may participate in the development of the reproductive system in parasites. These findings agreed with the results obtained through gene-expression detection of worm-dissected organs. Moreover, *SPC25* was also observed in tissues other than reproductive organs in *S. japonicum*. Consistent with the experimental results, the single-cell sequencing data of *S.japonicum* also showed that the *SPC25* gene has a higher expression level in germline stem cells and tegument cells (data unpublished). Based on the specific location of *SPC25* transcription in *S.japonicum*, we next examined the morphology of reproductive organs in the dsRNA-treated parasites.

### *SPC25* silencing leads to morphological changes of reproductive organ in *S. japonicum*

**Figure 4:**
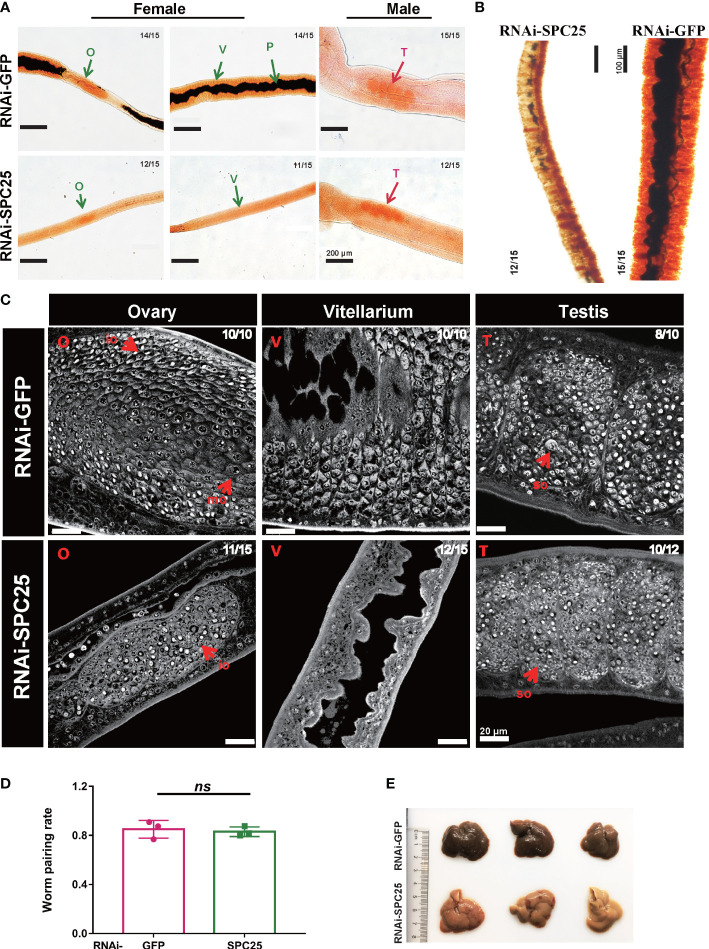
Morphological analysis of the reproductive organ in recovered RNAi-SPC25 worms at 30 dpi. **(A)** Microscopy images of carmalum staining of *S. japonicum*. **(B)** Microscopy images of Fast Blue B staining female worm. Positive Fast Blue B staining indicates mature vitelline cells. **(C)** Confocal laser scanning micrographs documenting morphological changes in the reproductive organs of RNAi-SPC25 group and control group worms. T, testis; O, ovary; V, vitellarium; P, intestinal pigment deposition; mo, mature oocytes; io, immature oocytes; so, spermatocytes. **(D)** The pairing rates of RNAi-SPC25 and RNAi-GFP worms. Each group had three biological replicates, and the data were shown as means ± SEM. **(E)** Liver receiving from the mice injected with dsGFP and dsSPC25. X/Y: number of worms with the same phenotype/number of worms analyzed in one biological replicate. ns, not significant, t-test.

In the RNAi-GFP group, the reproductive system exhibited a developmentally mature vitellarium and ovary, and uteruses were filled with eggs in the female worms; the male testicular lobes were loaded with mature spermatocytes. Compared with these, *SPC25* silenced parasites had less pigmentation in intestinal tissue, dysplasia in the vitellarium, ovary and testes, and lacked mature germ cells (**Figure 4A**). The Fast Blue B staining results showed that female worms failed to get mature vitellarium in the RNAi-SPC25 group (**Figure 4B**).

The laser scanning confocal microscopy image provided a qualitative analysis of the changes at the cellular level due to *SPC25* knockdown. The size of the testicular lobes displayed a significant reduction, accompanied by rare mature spermatogonia and spermatocytes in the testicular lobes of male worms from the *SPC25* dsRNA-treated group (**Figure 4C**). Moreover, most RNAi-SPC25 female parasites failed to get mature vitellarium and ovary, which has significantly decreased diameters of ovaries or even no ovaries, scantily organized vitelline cells within the vitelline lobes and rare eggs in the uterus (**Figure 4C**). Moreover, we also found that inhibiting the expression of *SPC25* reduced the pathological damage to the host’s liver without affecting the pairing of male and female worms (**Figures 4D, E**). Mice in the RNAi-SPC25 group had less egg deposition and granuloma in the liver (**Figure 4E**).

These results about worm morphology provide evidence that *SPC25* has a high probability of participating in sexual maturation in schistosome development. Therefore, we next explored the effect of silencing *SPC25* on oviposition in mature *S.japonicum*.

### SPC25-knowdown impairs reproductive system of adult parasites *in vivo*

While defining the effects of *SPC25* suppression on the adult worm oviposition, we carried out RNA interference on the parasite from 26 dpi to 42 dpi (**Figure 2A**). *SPC25* was also knockdown in the adult stage of *S. japonicum* (**Figure 2B**). Following hepatic portal perfusion, we only recovered about 50% of RNAi-treated worms initially infected (**Figure 2C**), suggesting RNAi-SPC25 parasites cannot maintain infection *in vivo*. Measurement of the control and RNAi-SPC25 groups’ worm lengths confirmed that the exhaustion of *SPC25* could prevent the further development of adult female worms and had almost no effect on the normal development of adult male worms. In particular, the further growth of adult females was significantly delayed. Compared with the control group, the length of the females in the RNAi-SPC25 group was shortened by 4.09 mm. Inhibiting the expression of *SPC25* in mature males had little effect on the development of the worms (**Figures 2D, E**).

**Figure 5:**
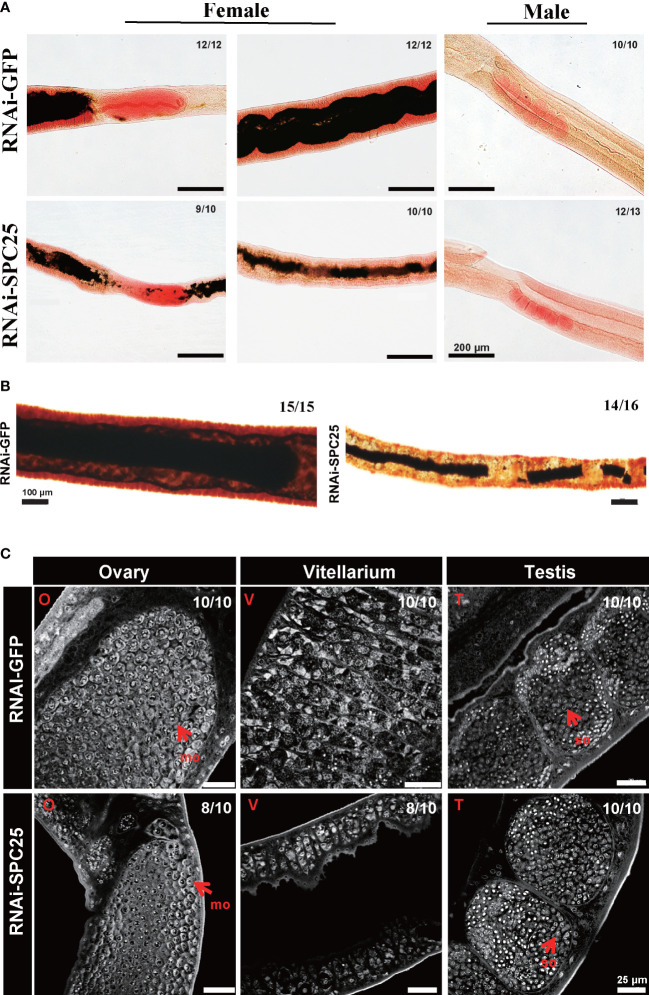
Morphological analysis of the reproductive organ in recovered RNAi-SPC25 worms at 42 dpi. **(A)** Microscopy images of carmalum staining of *S. japonicum*. **(B)** Microscopy images of Fast Blue B staining female worms. Positive Fast Blue B staining indicates mature vitelline cells. **(C)** Confocal laser scanning micrographs documenting morphological changes in the reproductive organs of RNAi-SPC25 group and control group worms. T, testis; O, ovary; V, vitellarium; P, intestinal pigment deposition; mo, mature oocytes; io, immature oocytes; so, spermatocytes. X/Y: number of worms with the same phenotype/number of worms analyzed in one biological replicate.

To further analyze the morphological changes of adult worms caused by *SPC25* knockdown, the parasites were stained with Fast Blue B staining and Mayer’s carmalum. Compared with controls, we found that adult worms treated with dsSPC25 presented morphologic changes, which mainly reflect the severe degeneration of the female worm’s original mature reproductive organs (vitellarium), accompanied by the size decrease or even loss of vitellarium cells (**Figures 5A, B**). Consistent with our observations of *S. japonicum* morphologic changes, the parasite images from confocal microscopy also displayed a severe impairment on the adult female reproductive system, including a high density of immature cells within the ovary and lower density cells within the vitellarium, compared to the RNAi-GFP group (**Figure 5C**). Whereas, no morphological alterations were observed in the testes of adult survival male worms (**Figure 5C**). These results showed clear evidence that *SPC25* was crucial for maintaining the normal development and morphology of the reproductive organs of female worms.

The degeneration of female reproductive organs means the reduction of oviposition and weakening pathological damage to the host. Thus, we further explored the reproduction and pathogenicity of RNAi-SPC25 worms.

### SPC25-knockdown worms attenuated egg viability and pathogenicity

**Figure 6:**
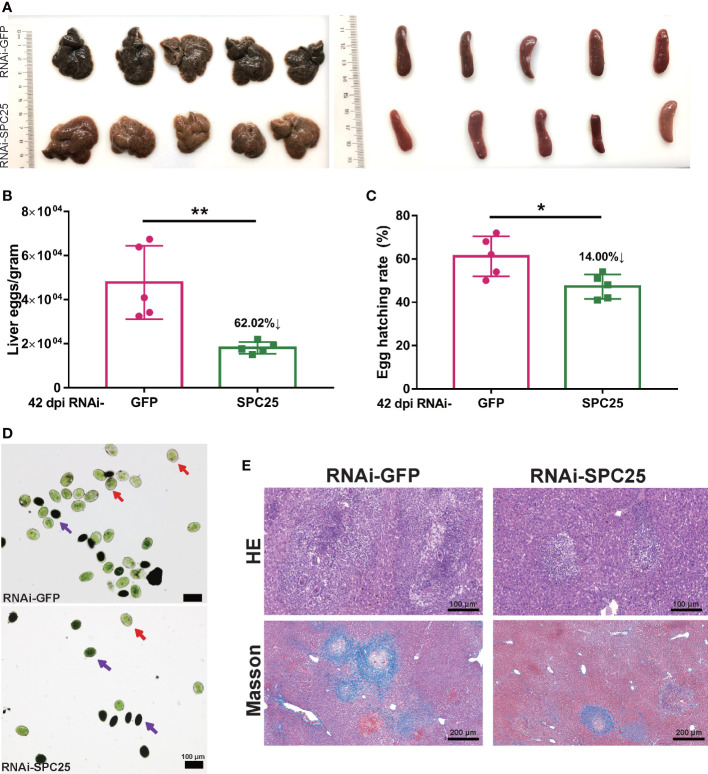
Examination of fecundity and the pathogenicity of RNAi-SPC25 worms. **(A)** Representative livers and spleens from the five mice per experimental group. **(B)** Egg number of liver per gram from RNAi-GFP and RNAi-SPC25 group mice on the 42 dpi. **(C)** Hatching rates of the eggs from the different groups of mice liver. Each group had five biological replicates, and the data were shown as means ± SEM. **(D)** Acridine orange staining of *S. japonicum* eggs. Red arrows indicated mature eggs, and purple arrows indicated immature eggs. **(E)** Histological assessment of mouse liver by HE and Masson staining. Each group had five biological replicates, the images shown were representative experimental results of one mouse. *p < 0.05, **p < 0.01, t-test.

In the course of studying the effects of *SPC25* knockdown on egg production and host pathogenicity, we selected a mouse model infected with SPC25-silenced *S.japonicum* for 26-42 days (**Figure 2A**). The experimental results showed that mice injected with dsSPC25 had insignificant hepatosplenomegaly and fewer granulomas on the liver than controls (**Figure 6A**). The statistical results of the number of oviposition showed that after infection with RNAi-SPC25 worms, the number of eggs in the mouse liver was reduced by 62.02% compared with the control group (**Figure 6B**). We previously demonstrated that *SPC25* expression inhibition results in abnormal ovaries and vitellarium function, leading us to hypothesize that egg motility was impacted. Observing the egg hatching rate showed that nearly 52.80% of eggs laid by RNAi-SPC25 worms were immature, and about 61.20% of eggs from the control group could develop into miracidia (**Figure 6C**). Results from acridine orange fluorescence staining of eggs were consistent with the hatching rate, which showed a higher proportion of mature eggs in the mouse liver of the RNAi-GFP group than the RNAi-SPC25 group (**Figure 6D**). As we all know, viable eggs of schistosomes were capable of the formation of granulomas. The weakening of oviposition ability and egg activity means the host’s pathological damage caused by *S.japonicum* may be alleviated.

Examination of histological sections from the livers of mice confirmed that eggs of the RNAi-GFP group were capable of generating a larger size of egg-induced granuloma, whereas very small egg-induced granuloma was observed in the RNAi-SPC25 group. According to Masson staining, the RNAi-SPC25 group had significantly less hepatic fibrosis (collagen fibers in blue) than the control group. Moreover, in the RNAi-SPC25 group, the region of hepatic fibrosis created by a single egg was smaller than in the RNAi-GFP group (**Figure 6E**). Thus, these results also identified our hypothesis that the *SPC25* suppression of *S. japonicum* could reduce the pathological damage to the host.

### Inhibition of *SPC25* activity results in reduced pairing rate of *S. japonicum in vitro*

**Figure 7:**
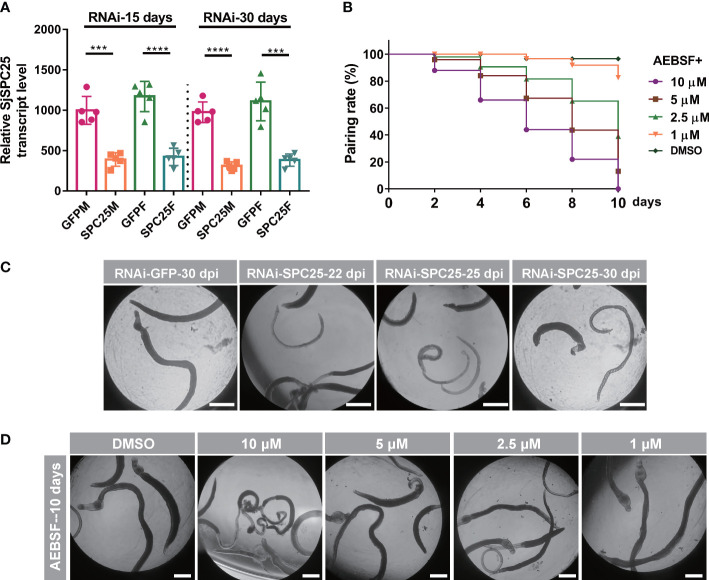
Worm activity analyses of inhibition of *SPC25* function *in vitro*. **(A)** Efficacy assessment of dsRNA-mediated *SPC25* knockdown of worm collected on the 15 dpi and 30 dpi. **(B)** Effects on worm motility of dsSPC25 treatment. All experiments were performed in triplicate with five biological replicates, and each group’s data were shown as means ± SEM.**(C)** The pairing rate of the inhibitor ABSEF treated worms *in vitro.* **(D)** Detection of visible abnormalities in ABSEF treated worms. Three independent biological replications were performed. And the well had six fresh pairs of worms for each biological experiment. ***p < 0.001, ****p < 0.0001, t-test.

Adult worms were incubated with either the inhibitor or dsRNA to determine whether inhibiting *SPC25* activity had an effect on parasite viability *in vitro*. The efficiency of *SPC25* knockdown was detected at 15 days and 30 days using qRT-PCR. We found that *SPC25* dsRNA constructs exerted effective transcript level suppression, ranging from 60% to 64% at 15 days and from 65% to 68% compared to controls at 30 days (**Figure 7A**). After 22 days of treatment, parasites were unable to attach to the surface of cell culture dishes. Partially dsRNA-treated female and male worms became unpaired and had darker bodies after 25 days of culture *in vitro* (**Figure 7B**). During this process, we observed that the silencing of *SPC25* led to tissue and intestinal edema, muscular hypercontraction. At this time, worms started manifesting muscular and gut vacuolation. After 30 days, some worms showed severe damage and even death, like darker whole bodies (**Figure 7B**).

The 4-(2-aminoethyl)-benzenesulfonyl fluoride HCl (AEBSF) is an irreversible serine protease inhibitor that inhibits the protease activity of SPC elements. Moreover, this inhibitor also prevented *Toxoplasma gondii* invasion of the host cells by specifically affecting parasite targets in a dose-dependent manner (Conseil et al., 1999). Subsequently, adult worms were exposed to four different concentrations (10 µM, 5 µM, 2.5µM and 1 µM). And 2.5 µM was the dose at which more than half of the paired worms were no longer paired, but the viability of male worms was not significantly affected (**Figure 7C**); 10 µM was the dose at which the worms significantly reduced their viability and were no longer attached to the cell culture dish, and some worms died at least four days after inhibitor exposure (**Figure 7D**). Consistent with the results of this experiment, when the *SPC25* gene interfered *in vitro*, the worms would weaken the activity and no longer pair on the 22 days, which was consistent with the shape of the worm body after treatment with the inhibitor. The results confirmed that inhibiting the function of *SPC25* could prevent the pair and reduce the viability of worms cultured *in vitro.* However, during experiments of injecting dsRNA or ABSF into mice, it was not found that the pair rate of male and female worms was affected (data not shown).

## Discussion

Schistosomiasis, a chronic and potentially lethal tropical disease, is caused by a large number of eggs output from mature female worms deposited in the host liver and intestinal wall, wherein they trigger granuloma formation and other pathological damages (Costain et al., 2018; Hotez et al., 2019; Abou-El-Naga, 2020). If the parasites could be killed or deprived of their ability to egg production in the host, both host pathological disruption and disease transmission will be prevented (deWalick et al., 2012). In the process of analyzing the transcriptome data of *S. japonicum*, we found a group of exciting genes (signal peptidase complex, SPC) that possess a unique expression pattern that is highly expressed in the developmental stages of the reproductive organ. Meanwhile, recent studies have shown that *S. japonicum* had some genes silenced that were specifically located in the reproductive organs, which significantly reduced egg production (Beckmann et al., 2010; Wang et al., 2017; Cheng et al., 2019). Moreover, studies of SPC-related genes in other organisms have confirmed that they are crucial to the reproduction and survival of organisms from yeast to mammals. In *Drosophila*, *Spase12* depletion is embryonic lethal and can disrupt the development of all tissues tested, including the eye, wing, leg, and antenna (Haase Gilbert et al., 2013). In addition, *SPC18* contributes to progression *via* TGF-α secretion in gastric cancer, and it is considered an important drug target for cancer treatment (Oue et al., 2013; Oue et al., 2014). Despite the apparent importance of the SPC for multicellular organisms, especially organs or tissues with rapid cell proliferation properties, *in vivo* studies investigating its function in *S. japonicum* have yet to be reported. Thus, we attempt to employ the RNA interference (RNAi) platform previously established in our laboratory to define SPC-related gene function (Li et al., 2018; Wang et al., 2022).

In the present study, RNAi-mediated silencing of SPC-related genes in schistosomula showed that SPC knockdown not only caused growth retardation but also reduced the survival rate of the parasites. Interestingly, suppressing the expression of the four SPC components resulted in differences in the worm reduction rate and length shortening. These results may be due to differences in the inhibitory effects of dsRNA on target genes, and of course, it does not rule out differences in the importance of the four elements for SPC (Mullins et al., 1996; Paetzel et al., 2000). After inhibiting SPC18 expression, the worm reduction rate reached 86.28%, and the surviving worms were small. Since the SPC elements had the same expression pattern and location and the expression changes of the remaining elements were consistent after knocking down *SPC25*, we speculate that the elements have the same biological functions. Meanwhile, the experimental samples obtained from the RNAi-SPC18 group were not conducive to our subsequent gene function research, so we integrated the data of worm length and worm reduction rate and then selected the *SPC25* gene for more detailed functional research.

*SPC25* expression profiles at different developmental time points and in the reproductive organs of *S. japonicum* showed that *SPC25* might play an important role in parasite reproductive organs. In addition, the WISH results showed that *SPC25* has a high expression level in the ovary and vitellarium of female worms, which is consistent with the developmental stage of the ovary and vitellarium in the female worm. These findings implied that inhibiting *SPC25* expression may impact the growth of female reproductive organs. As a result, we examined the morphology of the infected worms’ reproductive organs. Moreover, previous studies have shown that the signal peptidase is essential for synthesizing secreted soluble proteins and membrane proteins in bacterial and eukaryotic cells. This enzyme was thought to be a potentially valuable drug target (Paetzel et al., 2000; Harbut et al., 2012; Chen et al., 2018). As a result, effective anti-signal peptidase inhibitors may offer a new class of antiparasitic medications that effectively halt the development of the reproductive system or eradicate the schistosome worm.

In the present study, RNAi-mediated silencing of *SPC25* in schistosomula showed that the *SPC25* knockdown not only caused growth retardation but also reduced the survival and fecundity of the parasites. Growth delay leading to a lack of adequate resistance of the worm to host clearance may be a significant factor in worm mortality. The *SPC25* was significantly overexpressed in the tegument of male worms, and *S. japonicum* tegument changes are the primary adaptative mechanism for escaping the host’s immune response in several studies (Abath and Werkhauser, 1996; Castro-Borges et al., 2011; Sotillo et al., 2015; Perally et al., 2021). Thus, for the RNAi-treated schistosomula, the inability to settle down might be linked to their immature tegument at 30 dpi (Brouwers et al., 1999). Collectively, these results contributed to our hypothesis that *SPC25* is associated with the normal development of schistosomula *in vivo*. However, it is unclear whether the reason for the attenuated fertility of parasites is the growth retardation or the destruction of reproductive organ function for the RNAi-SPC25 worm. Although the reproductive systems of female parasites showed conspicuous morphological changes, this is not enough to elucidate the association between *SPC25* and egg production.

In order to seek the answers to these questions, we conducted RNA interference on the adult parasites. Surprisingly, following hepatic portal perfusion from mice injecting *SPC25* dsRNA, we only recovered parasites partly compared with the infected initially. Due to the adult worm possessing complete teguments, the reason for the death of the RNAi-treated parasite might not be similar to that of the schistosomula.

One reason might be that the absence of some excretory/secretory (ES) proteins controlled by *SPC25* results in the disability of regulating host immune response, which causes an enhancement of host immune clearance (Duvaux-Miret et al., 1992; Rezende et al., 2004); A possible scenario is that the accumulation of misfolded proteins in the cells caused by the knockdown of *SPC25* kills the adult worm (Meyer and Hartmann, 1997; Haase Gilbert et al., 2013; do Patrocinio et al., 2020). Of course, more evidence is required to support this hypothesis. In seeking answers to the mystery of worm death, we found that RNAi-SPC25 leads to a higher death proportion in adult worms than schistosomula and is not fatal for the parasite *in vitro* culture. Therefore, the death of SPC25-knockdown worm may be partly dependent on the host immunity system.

Another result worth noting is that the reproductive organs of female survival worms appeared to be degenerate, but the testes of male survival worms did not find a distinct alteration. Further evidence about its relationship was presented by staining and confocal images, which showed the exhaustion of *SPC25* mRNA-induced disruption in the original, well-developed reproductive organs of the adult female worm. The vitellaria was deprived of orders and regular shapes and presented as a deficiency of reproductive cells or some empty cavity. These deformities caused a significant reduction in the number of eggs laid by the worm. Moreover, the pathological damage to the host was alleviated due to a dramatic decrease in egg deposition in the host’s tissues.

However, why was the morphology of RNAi-SCP25-treated adult males not affected? There may be two reasons for this problem: One cause is that the expression of *SPC25* is low in the adult male worm, having a specific resistance to interference, or the knockdown level did not cause abnormal morphology, while the other explains that the existing experimental methods are not enough to detect the phenotypic change in worms. Given the specific phenotype of reproductive organs and survival rates of RNAi-treated worms, we reasoned that *SPC25* is essential for maintaining the normal parasitism and fecundity of parasites, at least for the female worm.

Except for the number decreases, the egg granuloma in the mouse liver has shown a contraction in size. The decrease in egg burden causes a relatively mild immune response, which may contribute to the reduction in granuloma size (Li et al., 2021). Besides, the egg viability might be responsible for introducing smaller granulomata in the *SPC25* exhaustion group. So, our attention was switched to the study of egg vitality. Consistent with our hypothesis, we found that most eggs laid by RNAi-SPC25 female worms lost the ability to give rise to miracidia. Moreover, the mature eggs may be produced by female worms before treatment with *SPC25* dsRNA. Because the size of liver granulomas caused by dead or immature eggs is usually smaller than mature eggs, these results indirectly confirmed the results related to the egg hatching rate (Shen et al., 2017; Forson et al., 2019; Takaki et al., 2020). Thus, the attenuated pathological damage to the host caused by RNAi-SPC25 may be attributed mainly to two aspects: on the one hand, the reduced number of eggs and/or worms decreases the immune stimulation of the host; on the other hand, the impaired reproductive systems of female worms cause reduced egg activity. Whether one factor or a combination of factors plays a role in lessening the pathological damage of *S. japonicum* eggs for the host, this result is valuable to prevent the transmission of schistosomiasis.

ABSEF (2.5-10 M), a serine protease inhibitor, dramatically resulted in no longer paired male and female parasites, indicating a decrease in parasite vitality. Compared with adding ABSEF, inhibiting the expression of *SPC25* in cultured worms for a short period did not achieve the goal of affecting the viability and pairing of worms *in vitro* culture. Most likely, the inhibitor affects the activity of other serine proteases rather than only preventing the SPC element from performing its function. We will look for more specific small molecule inhibitors of SPC components for further experimental investigation. In addition, mice injected with the inhibitor ABSEF did not gain protection against schistosomiasis. Further experiments will be performed to explain this result.

## Conclusion

In this study, we characterized the SPC biology function, which is proven to play an indispensable role in the growth and survival of Schistosoma *in vivo*. The exhaustion of *SPC25* mRNA can deprive the ability of fecundity, survival, and egg vitality for parasites. Moreover, the inhibitor ABESF can be used as a candidate small molecule drug against schistosomiasis. More importantly, our finding provided a new strategy to research the gene function associated with fecundity in the definitive host and contributed to revealing a novel class of molecular drug target candidates against schistosomiasis, providing new therapeutic opportunities.

## Data availability statement

The original contributions presented in the study are included in the article/**Supplementary Material**. Further inquiries can be directed to the corresponding author.

## Ethics statement

The animal study was reviewed and approved by Ethics Committee of the National Institute of Parasitic Diseases, Chinese Center for Disease Control and Prevention in Shanghai, China (IPD2020-10).

## Author contributions

WH conceived the project, designed the experiments, and critically revised the manuscript. W-BY, FL and WZ performed the experiments and analyzed the data. W-BY drafted the manuscript. CT, AZ and C-SS helped in the implementation of the experiment. All authors contributed to the article and approved the submitted version. 

## Supplementary material

Supplementary Figure 1 Expression patterns of SPC elements in *S. japonicum*. **(A)** Dynamic expression profiles of *SPC12, SPC18, SPC22* and *SPC25* in transcriptome sequencing data (mean± SEM, n=3). **(B)** SPC elements boxplots showing gene expression levels in populations of *S.japonicum*.
