# Supplementary figures and images for: Inhibition of signal peptidase complex expression affects the development and survival of Schistosoma japonicum
Source: Front Cell Infect Microbiol. 2023 Mar 3;13:1136056. doi: 10.3389/fcimb.2023.1136056 (PMC10020623; doi:10.3389/fcimb.2023.1136056)

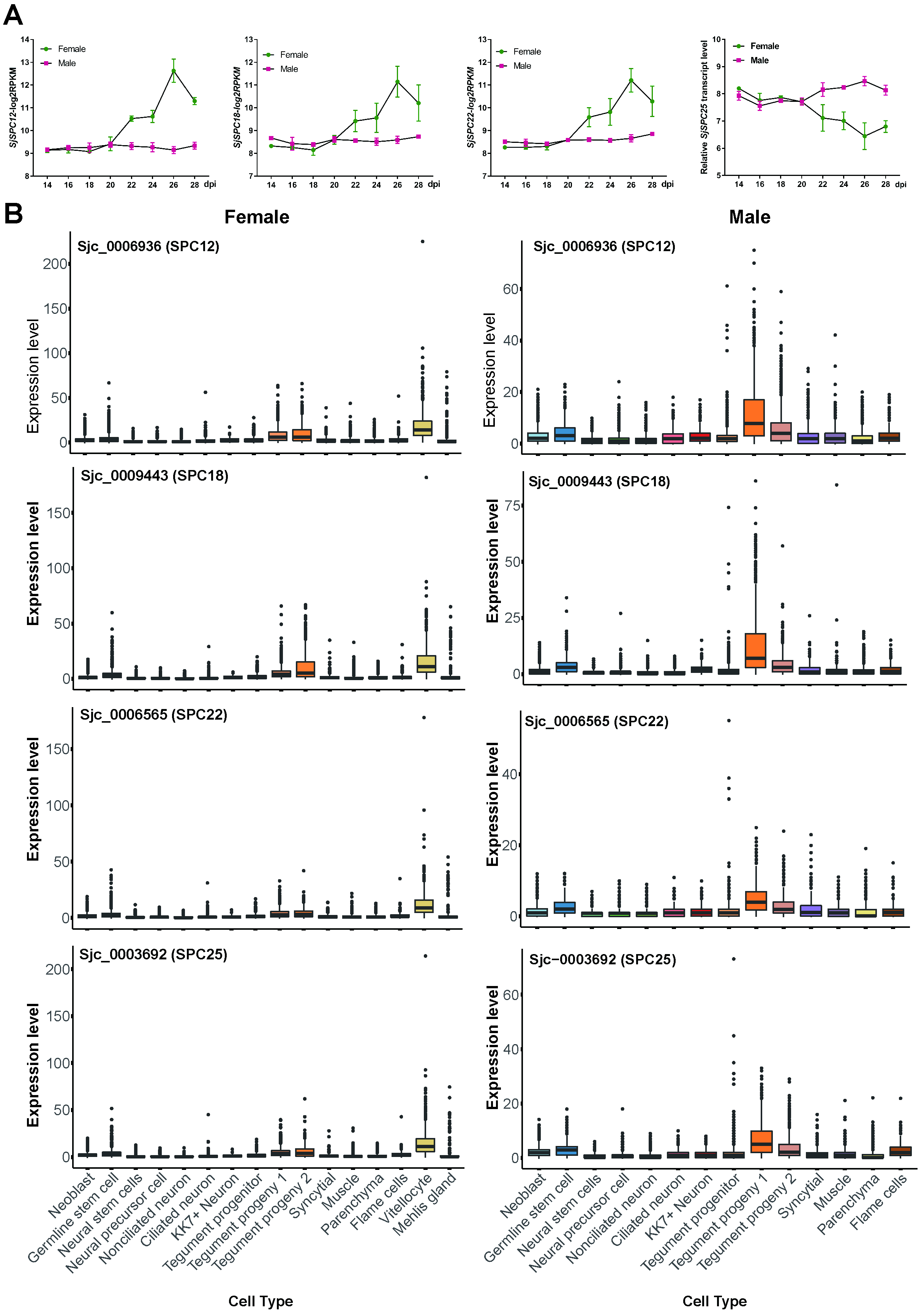

Supplement: Supplementary Figure 1 — Expression patterns of SPC elements in S. japonicum. (A) Dynamic expression profiles of SPC12, SPC18, SPC22 and SPC25 in transcriptome sequencing data (mean± SEM, n=3). (B) SPC elements boxplots showing gene expression levels in populations of S.japonicum. [file Image_1.tif]
